# ESAT-6 (EsxA) and TB10.4 (EsxH) Based Vaccines for Pre- and Post-Exposure Tuberculosis Vaccination

**DOI:** 10.1371/journal.pone.0080579

**Published:** 2013-12-12

**Authors:** Truc Hoang, Claus Aagaard, Jes Dietrich, Joseph P. Cassidy, Gregory Dolganov, Gary K. Schoolnik, Carina Vingsbo Lundberg, Else Marie Agger, Peter Andersen

**Affiliations:** 1 TB Vaccine Research, Department of Infectious Disease Immunology, Statens Serum Institut, Copenhagen, Denmark; 2 Adjuvant Research, Department of Infectious Disease Immunology, Statens Serum Institut, Copenhagen, Denmark; 3 Veterinary Sciences Centre, School of Agriculture, Food Science and Veterinary Medicine, University College Dublin, Belfield, Dublin, Ireland; 4 Department of Microbiology and Immunology, Stanford University School of Medicine, Stanford, California, United States of America; 5 Department of Microbiology and Infection Control, Statens Serum Institut, Copenhagen, Denmark; Colorado State University, United States of America

## Abstract

The ESX systems from *Mycobacterium tuberculosis* are responsible for the secretion of highly immunogenic proteins of key importance for bacterial survival and growth. The two prototypic proteins, ESAT-6 (EsxA from ESX-1) and TB10.4 (EsxH from ESX-3) share a lot of characteristics regarding genome organization, size, antigenic properties, and vaccine potential but the two molecules clearly have very different roles in bacterial physiology. To further investigate the role of ESAT-6 and TB10.4 as preventive and post-exposure tuberculosis vaccines, we evaluated four different fusion-protein vaccines; H1, H4, H56 and H28, that differ only in these two components. We found that all of these vaccines give rise to protection in a conventional prophylactic vaccination model. In contrast, only the ESAT-6-containing vaccines resulted in significant protection against reactivation, when administered post-exposure. This difference in post-exposure activity did not correlate with a difference in gene expression during infection or a differential magnitude or quality of the vaccine-specific CD4 T cells induced by ESAT-6 versus TB10.4-containing vaccines. The post-exposure effect of the ESAT-6 based vaccines was found to be influenced by the infectious load at the time-point of vaccination and was abolished in chronically infected animals with high bacterial loads at the onset of vaccination. Our data demonstrate that there are specific requirements for the immune system to target an already established tuberculosis infection and that ESAT-6 has a unique potential in post-exposure vaccination strategies.

## Introduction

*Mycobacterium tuberculosis* (*M.tb.*) remains a major threat to global health with an estimated 2 billion infected. The failure of the only currently available vaccine Bacillus-Calmette-Guerin (BCG) is evident by the 1.5 million lives lost each year [1]. Large efforts have been invested in the research and development of new and better vaccines, of which several are in various stages of clinical trials [2]. Although a large reservoir of *M.tb.* exists within the huge number of already tuberculosis (TB) infected individuals, the majority of vaccine candidates in development have been designed primarily for pre-exposure administration, either by replacing BCG using attenuated live mycobacteria or enhancing BCG with subunit vaccines. Nonetheless, preventing latently infected individuals from progressing to the contagious state of active disease is a crucial step in the eradication of *M.tb.* and will have a very pronounced influence on the global development of the TB epidemic as demonstrated by mathematical modeling [3]. 

The limited progress in developing efficacious post-exposure TB vaccines is due both to the difficulties associated with the establishment of robust and relevant animal models and the perceived safety risk (i.e. a Koch reaction) associated with post-exposure vaccines in clinical trials. In the 1950’s McCune and colleagues at Cornell University developed a model for studying mycobacterial persistence in the face of anti-TB drug treatment [4,5]. This so-called Cornell model has since it was introduced been exploited for studying latent TB infection and for the evaluation of post-exposure vaccines, making it the most rigorously characterized mouse model of latent TB. In the Cornell model, low levels of infection is induced by treatment with an antibiotic regimen post-challenge, after which the infection undergoes spontaneous or drug-induced relapse. Based on the Cornell model, several vaccine candidates, usually with prior documented pre-exposure activity, have been evaluated for protective efficacy against relapse when administered post-exposure. The outcome has mostly been disappointing and compared to the numerous reports of vaccines with preventive activity [2,6,7], only very few defined subunit vaccines have been demonstrated to have a significant protective effect in the demanding post-exposure animal model [8-11]. The reason for this difference is not clear but may relate to the distinct immunological milieu that *M.tb.* encounters in latently infected individuals where a hostile environmental ‘trigger’ changes of *M.tb.* characteristics including e.g. an altered gene expression pattern different from the one expressed in an immunologically naïve environment [12,13]. Hence, the requirements for an effective post-exposure vaccine to work in already infected individuals, with a pre-existing immune response and preformed granulomas, is potentially quite different from preventive vaccines as recently discussed elsewhere [14].

A recently published study introduced the H56 subunit vaccine, a fusion-protein incorporating Ag85B, ESAT-6 and Rv2660c, and described the high level of activity when administered post-exposure in the Cornell model [10]. In the present study, we dissected the background for this activity in detail by comparing pre-and post-exposure vaccine activity of H56, and the closely related vaccine H1 (Ag85B fused to ESAT-6) with the corresponding molecules (H28 (Ag85B-TB10.4-Rv2660c) and H4 (Ag85B-TB10.4), in which ESAT-6 has been replaced with TB10.4. Both ESAT-6 and TB10.4 are secreted by an export system referred to as Type VII secretion; ESAT-6 by ESX-1 and TB10.4 by ESX-3 [15]. ESX-1 is partly encoded from the region of difference 1 (RD1), important for virulence, the loss of which is the main attenuation factor involved in the generation of BCG [16,17]. ESX-1 secretion and consequently ESAT-6 export have been implicated in a wide palette of mycobacterial processes which contribute to pathogenesis, such as suppression of IFN-γ secretion by human T cells [18], blockage of phagosome-lysosome fusion and induction of host cell death [19]. Importantly, it has been demonstrated that ESAT-6 plays a direct role in producing pores in membranes of the vacuoles containing mycobacteria, facilitating mycobacterial escape from the phagosome to the cytosol [20]. ESAT-6 also appears to escape the infected host cell and promote granuloma formation through the up-regulation of metalloproteinase-9 on neighboring epithelial cells [21].

Although not nearly as well characterized as ESX-1, the ESX-3 system has been shown to be involved in iron acquisition [19] and recently a zinc binding site was identified in the TB10.4 protein itself, suggesting a direct involvement of this molecule in zinc ion acquisition [22]. Despite their different functions in *M.tb.* growth and virulence, ESAT-6 and TB10.4 have previously been demonstrated to share very similar immunological characteristics and are both strongly recognized during TB infection in animal models and by infected and diseased individuals [23-25]. 

Herein, we analyze a series of fusion-protein vaccines that differ only in their ESAT-6 and TB10.4 content and find that whereas both ESAT-6- and TB10.4-containing vaccines had prophylactic activity, only the ESAT-6-containing vaccines resulted in significant protection against relapse when administered post-exposure. 

## Results

### Development of a mouse model for post-exposure vaccination

**Figure 1:**
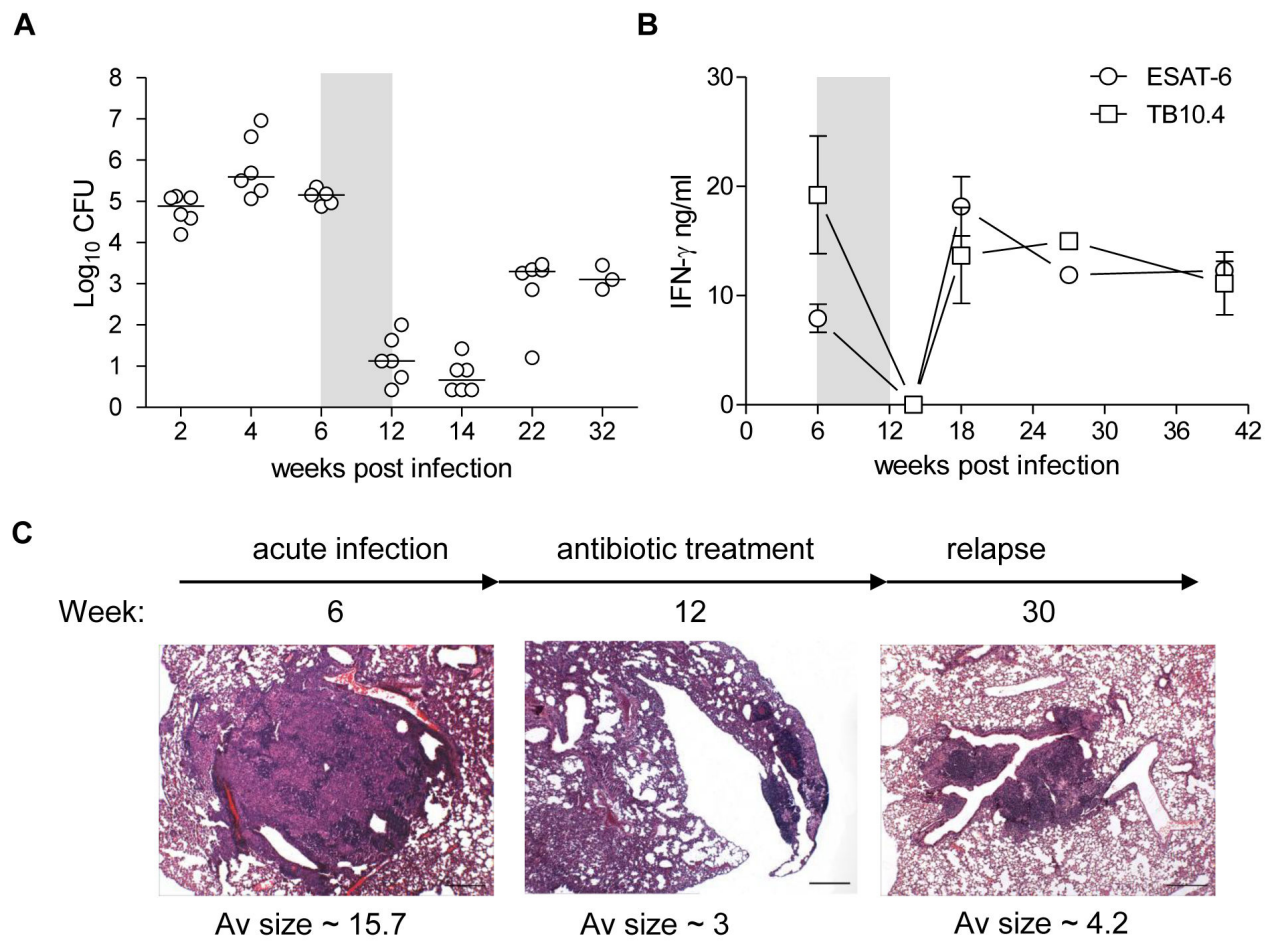
Mouse model of latent TB and relapse. (A) Groups of BALB/c x C57BL/6 (CB6F1) mice were infected by aerosol route with approximately 50 CFU of *M.tb*. Erdman. At week 6 post infection (p.i.) mice were given antibiotics in the drinking water *ad* *libitum* for six weeks (grey area) and allowed to spontaneously resume growth. Five-six mice (week 32 p.i. n=3) were sacrificed at various time-points during infection and lungs were harvested for determination of bacterial load during primary infection (week 0-6 p.i.), antibiotic treatment/latent phase (week 6-14 p.i.) and during the relapse phase (week 22-32 p.i.) and are expressed as log_10_ CFU. Data are shown as scatter dot plot and medians are displayed as a horizontal line for each time-point. (B) Lymphocytes were isolated at various time-points from perfused lungs and re-stimulated with ESAT-6 (open circles) and TB10.4 (open squares). Antigen-specific IFN-γ response was measured by ELISA. Data are shown as mean ± SEM of three independent experiments except for week 14 and 27 which are from one experiment. (C) Photomicrographs illustrating typical ‘acute’/primary infection (week 6 p.i.), antibiotic treatment/latent (week 12) and relapse (week 30 p.i.) phase lesions after aerosol infection with *M.tb*. Haematoxylin and eosin stain. Scale bar = 250 μm.

In order to evaluate vaccines for their activity post-exposure in animals infected with *M.tb.*, we developed and characterized an animal model that represents a modification of the previously published Cornell model [4]. Mice were exposed to *M*.*tb*. via the aerosol route. At week 6 post-infection (p.i.), the mice were treated with a combination of rifabutin and isoniazide provided in the drinking water for six weeks. Antibiotic treatment quickly resulted in significant reduction in lung bacterial load to levels below 2 log_10_ CFU and the bacterial numbers remained low for at least two weeks post-cessation of the antibiotic treatment (Figure 1A). After week 14, a spontaneous relapse of the infection was seen and the bacteria grew to between 3 and 4 log_10_ CFU measured at week 22 p.i. (10 weeks post-cessation of antibiotic treatment). The bacterial load was reflected in the IFN-γ response directed to both ESAT-6 and TB10.4 with responses after completed antibiotic treatment below detection levels and highly increased levels as the bacteria resumed growth (Figure 1B). 

We also examined the pathological changes in the animals during the course of infection. Six weeks post-infection when antibiotic treatment was initiated, relatively large granulomas were detected in the lungs (Figure 1C, acute infection week 6 p.i.). Although reduced in size, granulomas could still be identified following six weeks of antibiotic treatment (Figure 1C, antibiotic treatment, weeks 12 p.i.). By week 30 p.i. (Figure 1C, relapse), the increasing bacterial load due to bacterial regrowth was associated with multifocal un-encapsulated granulomas, frequently surrounding the bronchioles, consisting of sheets of closely apposed microvesiculated macrophages with infiltrating dense aggregates of lymphocytes.

### ESAT-6 but not TB10.4 containing fusion-protein vaccines prevent relapse of infection

**Figure 2:**
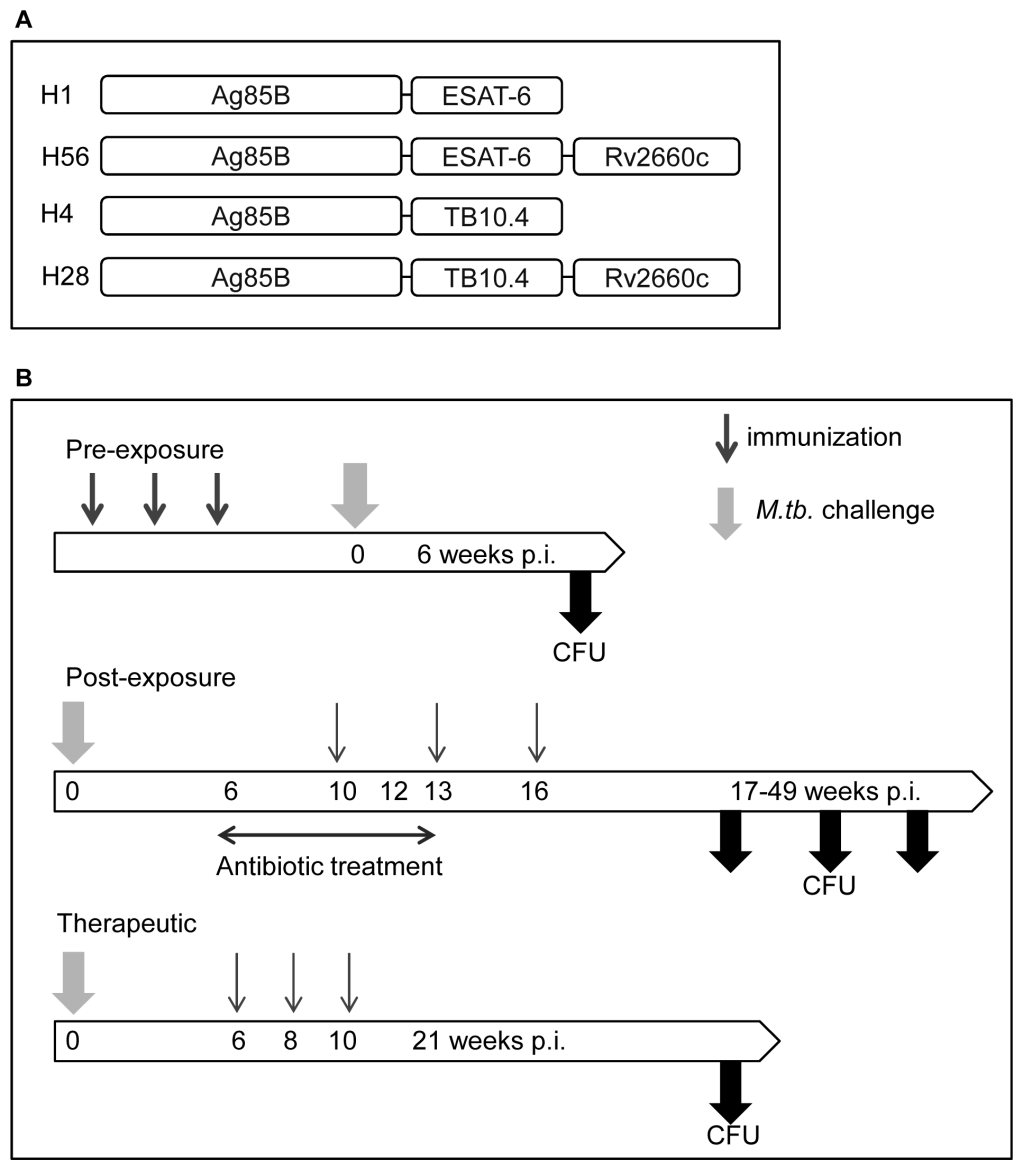
The vaccine constructs and animal challenge models. (A) A schematic overview of the multi-component vaccine constructs which include the ESAT-6-containing vaccines: H1 (Ag85B-ESAT-6) and H56 (Ag85B-ESAT-6-Rv2660c), and the TB10.4-containing vaccines: H4 (Ag85B-TB10.4) and H28 (Ag85B-TB10.4-Rv2660c). (B) Experimental setups for the pre-exposure, post-exposure and therapeutic models. **Pre-exposure** **model**: Mice receive three immunizations with a two week interval between each immunization. Six weeks after the last immunization, mice were aerosol challenged with 100 CFU of *M.tb*. Erdman. Six weeks post-infection (p.i.) mice were sacrificed and CFU levels monitored in the lungs. **Post-exposure** **model**: Mice receive an aerosol challenge with 50 CFU of *M.tb*. Erdman. After six weeks of infection, antibiotics are administered through the drinking water for a six week period. Vaccinations are given at week 10, 13, and 16 after infection. CFU levels in the lungs are monitored between week 17 and 49 p.i. **Therapeutic** **model**: Mice receive an aerosol challenge with 50 CFU of *M.tb*. Erdman. The mice receive three immunizations with two weeks interval (starting at week 6 p.i.), and the CFU levels in the lungs are monitored at week 21 p.i.

The ESX family members ESAT-6 and TB10.4 have previously been demonstrated to share very similar characteristics regarding immune recognition during infection and protective activity in preventive TB vaccines [26]. To further study the role of these antigens in TB vaccines for preventive and post-exposure administration we compared vaccines that differ only in these two components. The H56 vaccine contains Ag85B, ESAT-6 and Rv2660c [10], where H28, a very similar vaccine, differs only by the substitution of ESAT-6 with TB10.4 [27] (overview of all vaccine constructs and models can be seen in Figure 2A and B). 

#### Pre-exposure vaccination

**Figure 3:**
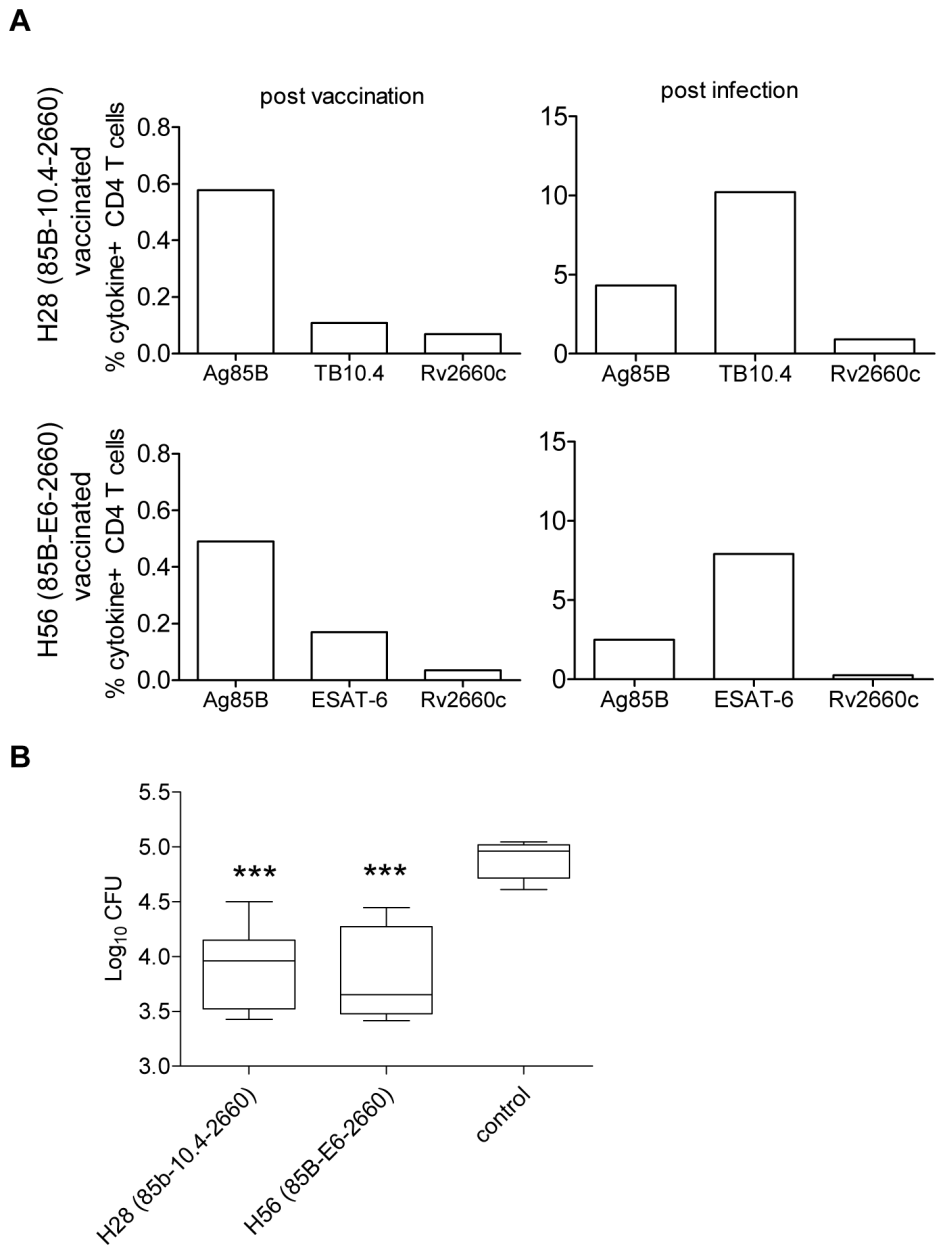
Prophylactic vaccination with H28 and H56 result in similar levels of protection. Groups of CB6F1 mice were vaccinated three times with two weeks interval with H28 or H56 formulated in CAF01 or with CAF01 alone. (A) Two weeks following vaccination with either H28 or H56, PMBCs (pooled from six mice) were stimulated with Ag85B, TB10.4, ESAT-6 or Rv2660c and antigen specific responses assessed by flow cytometry. The frequency of cytokine producing CD4 T cells expressing IFN-γ, IL-2 or both cytokines was determined by intracellular staining for cytokines (ICS). Lung lymphocytes (pooled from six mice) were obtained after six weeks of infection and the frequency of cytokine producing CD4 T cells determined by ICS. Data are representative of three experiments. (B) Protection was assessed at week 6 post infection by culturing lung homogenates and enumerating bacteria (expressed as log_10_). Data displayed are box and whiskers of six mice and minimum and maximum CFU levels. As a negative control, a group of mice received CAF01 only. Values marked with an asterisk are significantly different from naïve control animals as assessed by ANOVA and subsequent Tukey’s multiple-comparison test (***, *p*<0.001). The data are typical for several experiments as illustrated in Table S1.

To compare the immunogenicity of H28 (Ag85B-TB10.4-Rv2660c) and H56 (Ag85B-ESAT-6-Rv2660c), mice were vaccinated three times subcutaneously (s.c.) with the two fusion-proteins formulated in CAF01 cationic liposomes, previously reported to be an efficient delivery system for TB subunit vaccines [28]. Two weeks after the last immunization, peripheral blood mononuclear cells (PBMCs) were isolated and stimulated with the vaccine antigens and subsequently analyzed by intracellular staining (ICS) for IFN-γ and IL-2 production. Vaccine-specific CD4 T cells were detected in both H28 and H56 vaccinated animals (Figure 3A, left panel). The highest frequency of antigen-specific CD4 T cells was directed to Ag85B comprising 0.5-0.6 % of the CD4 T cell population in both vaccine groups. The TB10.4 response in H28 vaccinated mice and the ESAT-6 response in H56 vaccinated mice was lower compared to the Ag85B response comprising approximately 0.1% TB10.4 specific CD4 T cells in the H28 vaccinated group and about 0.2 % ESAT-6 specific CD4 T cells in the H56 vaccinated group. Six weeks following *M.tb.* challenge, pronounced immune responses directed towards TB10.4 in H28 vaccinated mice (10 %) and ESAT-6 in H56 vaccinated mice (8 %) were detected in the lung whereas the Ag85B specific responses comprised a smaller proportion of the CD4 T cell population (2-5 %) (Figure 3A, right panel). Although the Rv2660c response was measurable (H28: 0.9%, H56: 0.25%), the level remained much lower than the responses to the two ESX antigens. Hence, both H28 and H56 were highly immunogenic and promoted a response to the corresponding ESX antigen strongly recognized in the lung following infection. 

The vaccinated animals were tested for protection against *M.tb.* aerosol challenge by plating lung homogenates and culturing them for 2-3 weeks before counting bacterial colonies. Both H28 and H56 vaccination resulted in a significant reduction in the bacterial load compared to the control group (Figure 3B) and provided approximately 1 log reduction of bacterial numbers which is the level typically obtained by effective TB vaccines in this standardized model of preventive vaccination [7].

To further compare ESAT-6 and TB10.4-bearing vaccines, we expanded the comparison to cover the H1 (Ag85B-ESAT-6) [29] and H4 (Ag85B-TB10.4) [26] vaccines, and performed a series of challenge experiments. H4, H28, H1 and H56 pre-exposure vaccination resulted in similar levels of protection (in the range of 1-1.5 log reduction of CFU) (see Table S1). Thus, in agreement with previous publications, H1 and H56 promoted similar levels of protection in a short term challenge model [10] and this level was comparable to the TB10.4-based vaccines H4 and H28.

#### Post-exposure vaccination

**Figure 4:**
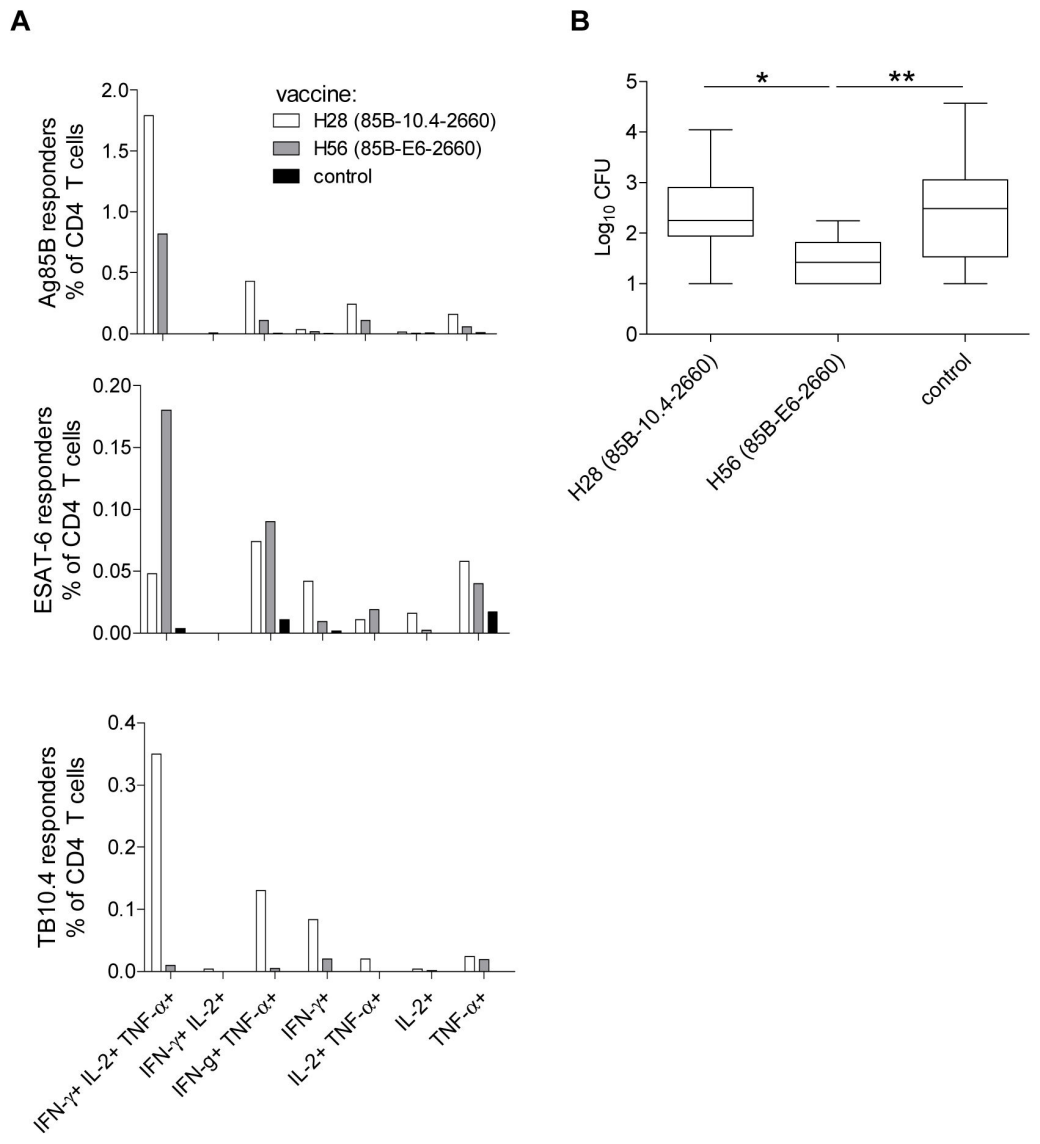
H56 but not H28 vaccination provides post-exposure protection against relapse. CB6F1 mice (n=12-18) were infected by the aerosol route and at week 6 post-infection (p.i.) treated with antibiotics given in the drinking water *ad* *libitum*. Mice were vaccinated three times s.c. with CAF01 alone, H28/CAF01 or H56/CAF01 at week 10, 13 and 16 p.i. (A) PBMCs (pooled from 16 mice) were obtained by submandibular bleeding one week after the last vaccination and the frequency and quality of antigen specific CD4 T cell responses were determined by six hour culturing of lung lymphocytes with Ag85B, TB10.4 or ESAT-6 from adjuvant control (black bars), H56/CAF01 (grey bars) and H28/CAF01 (white bars) animals. After culturing, cells were stained for CD4, IFN-γ, IL-2 and TNF-α, analyzed by ICS and subpopulations were divided according to their cytokine profile. Similar patterns of immune responses have been observed in two other experiments. (B) Protection was assessed at week 44 p.i. by culturing lung homogenates (n=12 for test groups and n=18 for control group) for 2-3 weeks at 37°C and enumerating the number of bacteria (expressed as log_10_). Data displayed are box and whiskers showing median values and minimum and maximum CFU levels. Values marked with an asterisk are significantly different from naïve control animals as assessed by non-parametric Kruskall-Wallis multiple-comparison test (**, *p*<0.01, *, *p*<0.05). Data is representative of two independent experiments, although individual H28 and H56 vaccine evaluations have been done in several other experiments as illustrated in Figure 6.

We continued by comparing H28 and H56 in the post-exposure animal model described above. Infected mice were vaccinated at week 10, 13 and 16 p.i. and immune responses were assessed one week after the last immunization (week 17 p.i.). At this time-point infection-driven immune responses in CAF01 vaccinated control animals were barely detectable (Figure 4A). This was in contrast to the response measured in the blood of H28 and H56 vaccinated animals, where vaccine-specific CD4 T cells were readily detected against Ag85B (in both groups), ESAT-6 mostly in the H56 vaccinated group and TB10.4 exclusively in the H28 vaccinated group. Rv2660c-specific responses were below the threshold of detection by ICS (0.05%) and did not differ between the H28 and the H56 vaccinated mice (data not shown). The Ag85B-specific CD4 T cells in both the H56 and H28 vaccinated group were primarily poly-functional co-expressing the three cytokines IFN-γ, IL-2 and TNF-α although both IFN-y+TNF-α+, IL-2+TNF-α+ and TNF-α+ CD4 T cell populations were also represented (Figure 4A). The cytokine profile of the ESAT-6-specific CD4 T cells in the H56 group and the TB10.4-specific CD4 T cells in the H28 group were similar and with the same distribution as the vaccine-driven Ag85B response. The small but still detectable ESAT-6 response in the H28 group (which does not contain ESAT-6) was different and dominated by an infection-driven effector IFN-y+TNF-α+ CD4 T cell population whereas TB10.4 responses remained negligible in animals not receiving a TB10.4-containing vaccine (H56 and un-vaccinated control animals). 

The vaccines were subsequently tested for their ability to inhibit the growth of *M.tb.* in the TB post-exposure model. Bacterial numbers was assessed at week 44 p.i. (Figure 4B). In agreement with previously published data, H56 post-exposure vaccination efficiently controlled bacterial growth, resulting in approximately ten times lower numbers of bacteria in these animals compared to the control group. In comparison, H28 vaccination did not prevent regrowth of the bacteria and the bacterial load was similar to the load measured in control animals. H56 vaccinated mice had significantly lower levels of bacteria compared to both control animals and H28 vaccinated animals and only 2 out of 12 animals had relapse of bacterial growth when vaccinated with H56, whereas 8 out of 12 mice in the H28 group had reactivated (defined as a bacterial load above 2 log_10_).

### Comparison of ESAT-6 and TB10.4; vaccine efficacy, T cell quality and gene expression

**Figure 5:**
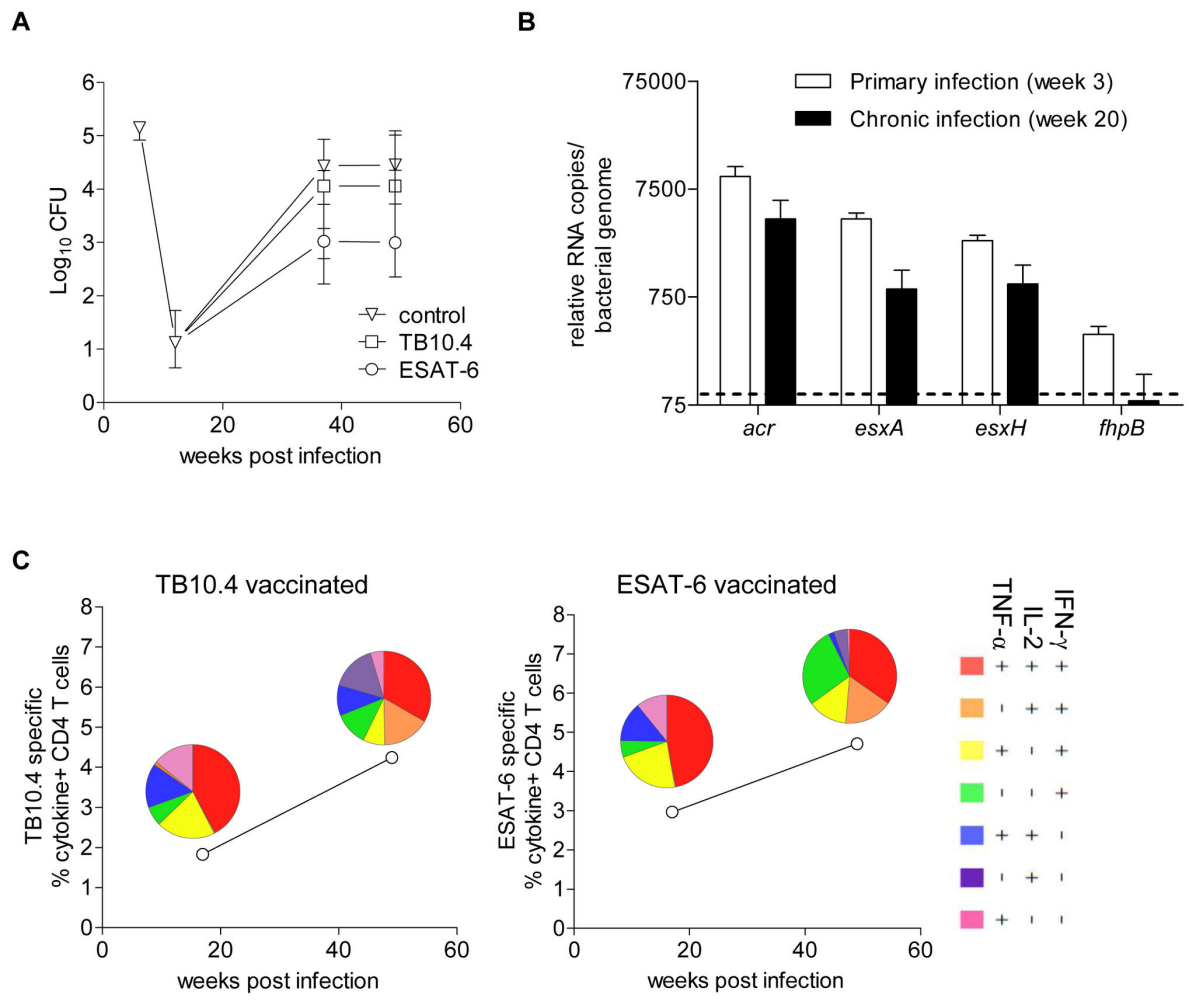
ESAT-6 and TB10.4; gene expression, immunogenicity and post-exposure vaccine efficacy. CB6F1 mice were infected, treated with antibiotics as described above and vaccinated with ESAT-6/CAF01 (n=7), TB10.4/CAF01 (n=7) or CAF01 alone (n=8) at week 10, 13 and 16 post infection (p.i.). (A) At week 37 and 49 p.i. mice were sacrificed, lungs harvested for assessment of protection by culturing lung homogenates at 37° C and enumerating the number of bacteria (expressed as log_10_). Log_10_ CFUs are shown for CAF01 only control animals (open triangles), TB10.4 (open squares) and ESAT-6 (open circles) vaccinated animals. Data are shown as median ± interquartile range and represent 6-8 individual animals. Evaluations of ESAT-6 and TB10.4 have been done in several experiments with similar outcome (the overall dataset is depicted in Figure 6). (B) *M.tb*. RNA was extracted from lung lobes of CB6F1 mice and used for real-time PCR to assess the gene expression level of the *acr, esxA, esxH* and *fhpB*. Data shown as relative transcript numbers (normalized data) and depicts the mean ± standard deviation (n=4). The dotted line denotes the level of detection at 100 relative RNA copies per bacterial genome. (C) Lymphocytes were extracted at week 17 and 49 p.i. from perfused lungs of six pooled mice, vaccinated with TB10.4 or ESAT-6. The cells were re-stimulated with their respective vaccine antigen and stained for CD4, IFN-γ, IL-2 and TNF-α for ICS analysis. Data is displayed as the total frequency of cytokine producing CD4 T cells (producing IFN-γ, IL-2, TNF-α or any combination of the three). The pie-charts represent the proportion of the subpopulation of responding CD4 T cells color-coded according to their cytokine profile.

As the only difference between H56 and H28 is the substitution of ESAT-6 with TB10.4, we decided to focus on the post-exposure vaccine activity of these two antigens individually instead of being part of fusion-proteins. We therefore compared the efficacy of ESAT-6 and TB10.4 post-exposure vaccination in controlling relapse at week 37 and week 49 p.i. (Figure 5A). ESAT-6 vaccinated animals had a lower bacterial load compared to the control group at both time-points (Δlog_10_: 1.41 and 1.46 at week 37 and week 49 p.i, respectively). Although the difference did not reach statistical significance for the individual time-points, ESAT-6 gave a statistical significant protection in the overall experiment (Mann-Whitney U-test: ESAT-6 vaccinated compared to control group: week 37 p.i. p=0.0541, week 49 p.i. p=0.1893, overall experiment p=0.0168). In contrast, TB10.4 vaccination only resulted in a very modest reduction of bacteria (approximately 0.4 log_10_ CFU compared to the control group), which was not significantly different from control animals at any time-point or for the experiment in total (TB10.4 vaccinated compared to control group: TB10.4 vs. control: week 37 p=0.3357 week 49 p=0.8665, overall experiment: p=0.4176). To address the possibility that the protective differences exerted by ESAT-6 and TB10.4 were a result of a different level of gene expression at different stages of the infection, we compared the expression of *esxA*, *esxH*, *acr* (alpha-crystallin) and *fhpB* (Ag85B) at week 3 and week 20 p.i. (Figure 5B). *acr* and *fhpB* was included for comparison as these genes have previously been reported to represent genes expressed either during early growth (*fhpB*) [13] or during hypoxia-induced stress (*acr*) [30]. The expression of *esxA* and *esxH* was found to be high and at comparable levels at week 3 p.i. At week 20 p.i. the onset of adaptive immunity and control of bacterial growth had resulted in a general down-regulation of gene expression but the relative expression of *esxA* compared to *esxH*, remained at the same level. The expression of *acr* was also down regulated at the late time-point but remained high in contrast to the expression level of *fhpB* which was below the detection level (100 relative RNA copies per chromosome) at week 20 p.i. The failure of TB10.4 post-exposure vaccination is therefore not a consequence of a specific down-regulation of *esxH* in the later stages of infection. Finally, we investigated if a difference in the quality of the vaccine-induced immune response could explain the protective differences after ESAT-6 and TB10.4 post-exposure vaccination. Lymphocytes from the lungs of vaccinated animals were obtained and re-stimulated with the homologous antigen preparation at two time-points, week 17 p.i. (1 week post last vaccination) and week 49 p.i. (week 33 post last vaccination). Approximately 2 % TB10.4 specific CD4 T cells were detected in TB10.4 vaccinated mice at week 17 p.i. and this frequency increased to approximately 4.5 % at week 49 p.i. (Figure 5C). The TB10.4-specific CD4 T cells consisted of about 35-50 % IFN-γ+IL-2+TNF-α+ CD4 T cells at the two time-points. A similar level of vaccine-induced response of a similar quality was observed in ESAT-6 vaccinated mice at the two time-points. Both the TB10.4 and ESAT-6 specific immune response in control animals (infection-driven) consisted for comparison primarily of IFN-γ+TNF-α+ CD4 T at both time-points (data not shown). Thus, the vaccine-induced response generated by TB10.4 was very similar to the ESAT-6 induced response both in terms of magnitude and quality i.e. the proportion of IFN-γ+IL-2+TNF-α+ CD4 T cells and differed markedly from the infection promoted response to these antigens.

### Large scale evaluation of post-exposure activity of ESAT-6 and TB10.4 containing vaccines

**Figure 6:**
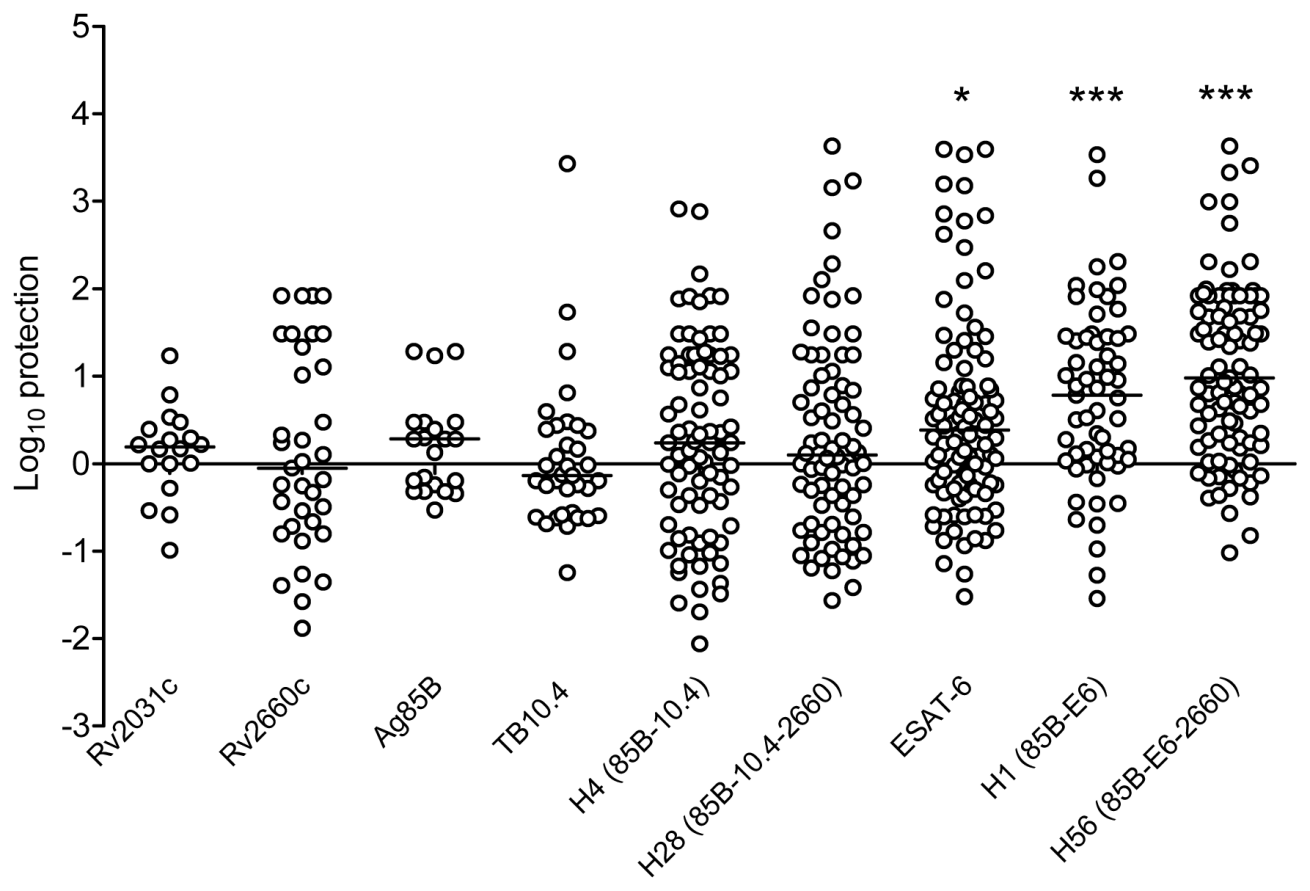
Meta-analysis of post-exposure vaccine efficacy. The post-exposure activity of various vaccines was tested in fifteen individual experiments including a total number of 726 mice. The data shown is a meta-analysis performed by determining the log_10_ protection for each individual experiment as follows: **log**_**10protection**_ **= median log**_**10**_ **CFU**_**controlgroup**_ **−log**_**10**_ **CFU**_**vaccinated animal**_ as described in the material and methods section. Data are displayed as single scatter plot with a black line indicating the median values. Values marked with an asterisk above ESAT-6, H1, and H56 are significantly different from the un-vaccinated control groups as assessed by the non-parametric Kruskall-Wallis multiple comparison test (***, *p*<0.001, **, *p*<0.01, *, *p*<0.05).

Given the surprising difference on the post-exposure activity of the H56 and the H28 vaccines (and the ESAT-6 and TB10.4), we proceeded by comparing the protective efficacy conferred by the individual components in these two constructs (Ag85B, TB10.4, ESAT-6 and Rv2660c), the two closely related fusion-proteins H1 (Ag85B-ESAT-6) and H4 (Ag85B-TB10.4) and the Rv2031c molecule. Rv2031c was included because of its widely accepted role as a latency-associated antigen; up-regulated under hypoxia [31] and preferential recognition in latently infected individuals [32]. To overcome the shortcomings related to intra-and inter-experimental variation, we normalized data for fifteen individual experiments conducted over 3 years within the Gates Grand Challenges GC12 Consortium (see materials and methods for details) and pooled the result into one large analysis based on data from 726 mice (Figure 6). The data from the pooled analysis is depicted as Δlog_10_ protection (i.e. the difference in bacterial loads in vaccinated animals compared to control animals in each individual experiment). Based on this analysis it was evident that vaccines giving significant levels of protection compared to the control group were the ESAT-6-containing vaccines H1, H56 as well as ESAT-6 administered alone. Post-exposure vaccination with H56 resulted in significantly better protection compared to all the other vaccine candidates included in the analysis, with the exception of H1. H56 in the pooled analysis resulted in a Δlog10 value compared to the control group of 0.9821 log compared to e.g. H28 which had a Δlog_10_ value of 0.09843. Thus, ESAT-6 containing vaccines have a unique capability to inhibit relapse of bacterial growth when given post-exposure which is not seen with any of the other antigens and vaccines, although these vaccines had prophylactic activity.

### ESAT-6 vaccination controls relapse of bacterial growth but does not reduce bacterial load in a chronic steady-state infection

Post-exposure administration of ESAT-6 based vaccines efficiently controlled the relapse of bacterial growth in the modified Cornell model and we continued by investigating if this activity had a broader application e.g. if ESAT-6 could be used therapeutically to influence the course of a chronic infection in mice. For this purpose, vaccination was administered in a model where the bacterial numbers had been stable throughout infection after establishment of adaptive immunity. Thus we wanted to determine if ESAT-6 post-exposure vaccination would perturb the host-pathogen equilibrium, in favor of the host, leading to a reduction in bacterial load in the steady-state scenario. 

**Figure 7:**
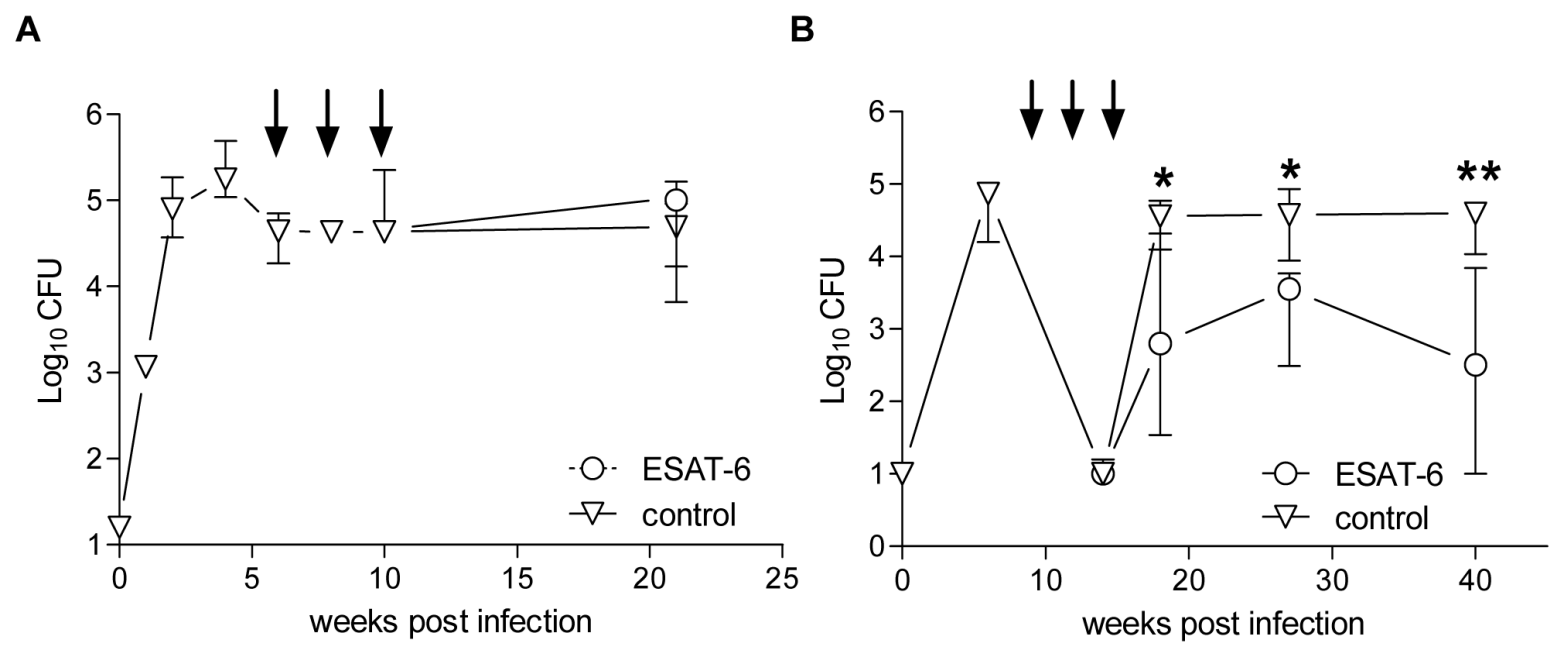
Comparison of therapeutic and post-exposure vaccine efficacy. (A) In the therapeutic model, CB6F1 mice were aerosolly infected and vaccinated three times with two weeks interval starting at week 6 post infection (p.i.) (black arrows) with ESAT-6/CAF01 (open circle) or CAF01 alone (open triangle). The therapeutic efficacy was assessed at week 21 p.i. Data shown are median values ± interquartile range week 0-20 from 3-8 individual animals and at week 21 p.i. n=7 for control animals and n=8 for ESAT-6 vaccinated. The two groups were compared with a Mann Whitney U-test and p<0.05 was considered significant. (B) In the post-exposure model, mice were infected for six weeks and then given antibiotics for six weeks, as described in materials and methods. Groups of mice were vaccinated with ESAT-6/CAF01 or CAF01 alone at week 10, 12 and 14 p.i. (black arrows) and protection assessed at week 18, 27 and 40 p.i. by culturing lung homogenates and enumerating the number of bacteria (expressed as log_10_). Data shown are median values ± interquartile range for the control group (open triangles, n=6) and ESAT-6 vaccinated group (open circle, n=6). Values marked with an asterisk are significantly different from naïve control animals as assessed by non-parametric Mann-Whitney U-test (**, *p*<0.01, *, *p*<0.05).

ESAT-6 was given to mice with a chronic infection characterized by a high bacterial load, approximately 5 log_10_ CFU, after reaching a steady-state condition following week 6 of infection (Figure 7A). Immunizations with ESAT-6 in CAF01 was given at week 6, 8 and 10 p.i. Therapeutic effect of ESAT-6 was evaluated at week 21 p.i. by plating and culturing lung homogenates. Post-exposure vaccination during a chronic infection did not reduce the bacterial load nor did it exacerbate disease compared to control animals (ESAT-6: 5.01 log_10_ CFU versus control 4.70 log_10_ CFU, respectively, p=0.8518). In agreement with the findings above, mice immunized with ESAT-6 in combination with antibiotic treatment in the modified Cornell model had a significant lower bacterial load compared to control mice at all time-points examined ranging from 1-2.3 log_10_ difference in median values (week 18 p= 0.0411, week 27 p= 0.0303, week 40 p= 0.0129) (Figure 7B). 

Thus, ESAT-6 post-exposure vaccination results in protection against relapse of bacterial growth when administered to mice with a low bacterial load at the time-point for vaccination. ESAT-6 vaccination does, on the other hand, not have a therapeutic effect when administered in mice with an ongoing chronic infection.

## Discussion

In the last 10 years there has been substantial progress in the TB vaccine field, with several vaccines in clinical trials. These leading vaccines are all designed as preventive vaccines and have been demonstrated in preclinical animal models to give varying levels of protection against *M.tb.* challenge [2,6,7]. This includes the MVA85A vaccine that recently failed to confer protection in a phase 2B efficacy trial in BCG vaccinated infants [33], a target population where it is clear in hindsight that the animal data supporting the performance of MVA85A are very limited [34]. Herein, our main question was whether vaccines with preventive activity in the mouse TB model would also protect against relapse when given post-exposure. The reason why this question is so urgent and relevant is because of the particular manifestation of the TB epidemic, where the population in dire need of a novel and efficient TB vaccine are latently infected with *M.tb.* and hence receive their vaccine post-exposure. Therefore, it is striking that although numerous studies have been published demonstrating protection against TB in animal models when given preventively [7], so far the examples of successful post-exposure vaccines in the literature are very limited [8-11].

Post-exposure vaccination against TB in a host which has co-existed for decades with *M.tb.*, adapted to survive the stress of adaptive immunity is obviously very different from preventive vaccination in a naïve host. The hypothesis that has been rigorously pursued by several groups is that *M.tb.* contained within granulomas undergo a transition from being metabolically active to metabolically inactive i.e. dormant with a concurrent shift in transcriptomic profile resulting in so-called latency antigens being expressed and available for recognition [35]. In this regard, it has been reported that BCG due to its attenuation and therefore early clearance, fails to drive a response to latency-associated antigens and this was suggested as one of its main weaknesses [36]. The search for vaccine candidates for a post-exposure vaccine has therefore been focused on identifying latency-associated antigens. To model these conditions, several *in vitro* models were established based on either oxygen or nutrient deprivation [31,37], and numerous antigens were identified including alpha-crystalline (Rv2031c) which is strongly up-regulated under conditions of hypoxia [30]. A large number of these antigens have been evaluated for their recognition during TB infection [38,39] and a very recent report demonstrates that many of these antigens are indeed recognized both by latently infected individuals and patients with active TB [40]. 

In spite of the extensive body of evidence that demonstrate recognition during infection, only a limited number of studies report protection in animal models promoted by a vaccine based on a late-stage expressed antigen. Dey and colleagues recently published a study where Rv2031c was used in an rBCG - DNA prime boost strategy that provided improved protection against TB [41,42], but as a stand-alone vaccine this molecule failed to protect [42]. We found that although post-exposure vaccination with Rv2031c induced vigorous IFN-γ responses (data not shown) and the gene encoding the Rv2031c protein (*acr*) was expressed at an extremely high level, no significant protection against relapse of bacterial growth in the Cornell model could be detected. Likewise, in the present study, Rv2660c, previously found to be upregulated in nutrient-deprived bacterial cultures [37] and included in the H56 fusion-protein vaccine [10], did not protect against relapse when given post-exposure.

In the current study, we found that the post-exposure activity of H56 (Ag85B-ESAT-6-Rv2660c) could largely be attributed to ESAT-6 because vaccination with TB10.4, H4 (Ag85B-TB10.4) or H28 (Ag85B-TB10.4-Rv2660c) did not result in equivalent protection. That H56 is more efficient than ESAT-6 alone is most likely due to the improved immunogenicity of this fusion molecule which results in improved responses to its constituents as was previously reported for vaccine fusions molecules [10,26]. ESAT-6 is not a latency-associated antigen based on the *in vitro* criteria described above but in agreement with previous publications [10,13], we found that ESAT-6 was expressed from the early acute to the late chronic phase of infection [10]. Hence, ESAT-6 has a constitutive expression profile that differs from e.g. Ag85B (encoded by *fhpB*) which is markedly down-regulated as the bacteria respond to adaptive immunity and increasing levels of IFN-γmediated macrophage activation [10,13]. In the TB murine model CD4 T cell responses to Ag85B are therefore a prominent feature in early infection but there is a clear correlation between the down-regulation of *fhpB* and decreasing levels of T cell responses to this antigen [13]. Despite a global down-regulation of genes expressed by *M.tb.* upon progression into the more chronic stages of infection, both ESAT-6 (*esxA*) and TB10.4 (*esxH*) expression remain relatively high (approximately 10-fold higher compared to *fhpB*) and both ESAT-6 and TB10.4 are therefore theoretically equally available for recognition by antigen-specific T cells during late/chronic disease [43]. 

Although this may help explain why ESX-1 based vaccines have the potential to work post-exposure under conditions where host adaptive immunity is already established, it does not explain the difference between the activity of ESAT-6 and TB10.4 containing vaccines as these two antigens are expressed at comparable levels. The response to both antigens is characterized by highly functional CD4 T cells expressing IFN-γ, IL-2 and TNF-α, as previously reported when the CAF01 adjuvant is used in preventive TB vaccines [44]. However, one obvious difference between ESAT-6 and TB10.4 is their role in bacterial patho-physiology. ESAT-6 is a key player in mycobacterial processes relating to virulence and forms the active component in a pore-forming complex that allows the bacteria to escape phagosomes [20,45,46], as well as the individual host cell [45]. There is also evidence that ESAT-6 is secreted and interacts directly with epithelial cells outside the *M.tb.* infected macrophage [21]. In contrast, the physiological role of TB10.4 is fundamentally different as this molecule is most likely involved in iron/zinc uptake, a process crucial to *M*.*tb*. because of a very low intracellular concentration of accessible ions [22]. One possibility is that the high level of protective activity after post-exposure vaccination with ESAT-6 and not TB10.4 relates to their functional differences, in particular that TB10.4 is expected to be released preferentially inside the infected cells, whereas ESAT-6 in addition to its intracellular secretion also exists and exerts its function in the extra-cellular milieu [21]. During co-evolution with its human host *M.tb.*, has developed strategies to evade elimination by the host adaptive immune response, mediated through inhibition of MHC class II molecule expression and antigen presentation [47]. One hypothesis is therefore that ESAT-6 specific CD4 T cells, promoted by post-exposure vaccination, bypass this general evasion strategy, because the ESAT-6 molecule, through escape from the interior of the host cell, is more readily available for immune recognition and importantly for uptake by non-infected and therefore functionally more capable antigen presenting cells within or in the vicinity of the granuloma. An alternative explanation could be that *M.tb.* escapes immune surveillance in the chronic phase of infection by varying the sequence of the *esxH* gene, thereby eliminating the epitope critical for the post-exposure activity of the TB10.4 based vaccines. The reason why this is a relevant consideration is based on the recent finding that human T cell epitopes derived from ESAT-6 (and most other known T cell antigens) were found to be hyper-conserved in clinical isolates whereas TB10.4 represent an outlier that contained a larger number of amino acid changes [48]. The possibility that the *M. tuberculosis* Erdmann challenge strain can mutate the *esxH* gene as an immune escape mechanism during long-term growth in mice is currently under evaluation. We do acknowledge the limitations of extrapolating from a murine model to human *M.tb.* infection. An inbred mouse strain with a restricted major histocombatibility complex (MHC) haplotype will narrow and bias the repertoire of antigens available for recognition compared to a genetically diverse human population. However, similar to our mouse model, both ESAT-6 and TB10.4 have previously been reported to be recognized at very similar levels in TB infected individuals [49]. Furthermore, we have demonstrated that both antigens provide protection when used as preventive vaccines. This excludes the possibility that the difference in post-exposure protection provided by TB10.4 and ESAT-6 containing vaccines is a simple consequence of genetic restriction of antigen recognition.

In the present study, we observed that while ESAT-6 had powerful activity when administered post-exposure, we did not observe protection when ESAT-6 was used for therapeutic vaccination i.e. in chronically infected mice. This could be a consequence of an overwhelmed immune system due to the presence of high bacterial numbers. However, given that these animals still mount a strong infection-driven CD4 T cell response (results not shown) another possibility could be a difference in ESAT-6 availability during the two conditions. In chronically infected mice bacterial numbers reach a steady-state, where a very limited replication is counterbalanced by immune mediated killing of organisms [50,51]. Increased levels of adaptive immunity and markedly reduced bacterial replication clearly results in a dramatic down regulation of mycobacterial gene expression including *esxA* (Figure 5B), which may prevent an efficient therapeutic vaccination. Our data therefore suggest that in a clinical setting, preventing relapse from a shortened chemotherapy treatment regimen may provide a better outcome than regular immuno-therapy immediately after the onset of chemotherapy.

An ESAT-6 containing vaccine may therefore have two potential applications which will target the vast reservoir of TB infected individuals; a post-exposure vaccine for populations where latent TB is widespread and an adjunct immuno-therapy to shortened chemotherapy treatment regimens for active TB. The clinical development of two ESAT-6-bearing subunit vaccines (H1 and H56) which includes testing in latently infected individuals will in the near future provide a clinical proof-of-concept testing, the outcome of which will be of critical importance for the feasibility of both applications.

## Materials and Methods

### Ethics statement

Experiments were conducted in accordance with the regulations set forward by the Danish Ministry of Justice and animal protection committees by Danish Animal Experiments Inspectorate Permits 2004-561-868 and 2009/561-1655, and in compliance with European Community Directive 86/609 and the U.S. Association for Laboratory Animal Care recommendations for the care and use of laboratory animals. The experiments were approved by the Statens Serum Institute animal ethics board headed by DVM Kristin E. Engelhart Illigen.

### Animals

Inbred female BALB/c x C57BL/6 (CB6F1) mice was obtained from Harlan Scandinavia (Allerød, Denmark). Mice were kept at the experimental animal facilities at Statens Serum Institut. Infected mice were housed in cages contained within laminar-flow safety enclosures (Scantainer; Scanbur) in a biosafety level 3 facility, and provided with irradiated food and filtered drinking water. 

### Cloning and purification of fusion proteins

The *rv2660c* gene was amplified by PCR from*M*.*tb*. H37Rv chromosomal DNA using either 5′-**GGATGTTCGCA**GTGATAGCGGGCGTCGAC-3′ or 5′-**ATGGGGCGGC**GTGATAGCGGGCGTCGAC-3′ as sense oligonucleotide and 5′-TTAGTGAAACTGGTTCAATCCCAGTATC-3′ as anti-sense oligonucleotide. Stop codon is underlined, and sequence overlaps to the *esxA* or *esxH* sequence are in bold. The amplified *rv2660c* gene was fused to *fhpB*-*esxA* or *fhpB*-*esxH* by overlapping PCR and inserted into the pDest17 expression vector (Invitrogen). *E. coli* BL21-SI cells (Invitrogen) harboring *fhpB, esxA, esxH, rv2660c, fhpB-esxA, fhpB-esxH, fhpB-esxH-rv2660c* or *fhpB-esxA-rv2660c* produced all the recombinant proteins as inclusion bodies. All proteins were purified according to the same protocol. Inclusion bodies were washed three times in 20 mM Tris-HCl pH 8.0, 100 mM NaCl, 1 mM EDTA, 0.1% deoxycholic acid and dissolved in 8 M urea, 100 mM Na2PO4, 10 mM Tris-HCl pH 8.0 (buffer A) before being applied to metal affinity columns (Clontech). Bound proteins were washed five times with two column volumes of buffer, alternating between 10 mM Tris-HCl pH 8.0, 60% isopropanol and 50 mM NaH2PO4 pH 8.0, before being eluted in buffer A supplemented with 200 mM imidazole. After inspecting for impurities, relevant fractions were pooled and dialyzed against 3 M urea, 10 mM Tris-HCl pH 8.5 and applied to anion-exchange columns (Pharmacia) washed with 5 column volumes and eluted using a linear NaCl gradient, from 0 to 1 M over 40 column volumes. Based on purity, fractions were pooled and dialyzed against 25 mM NaH2PO4 pH 8.0, 10% glycerol, 150 mM NaCl, 0.05% Tween20. Finally, protein concentrations (NanoOrange™ Protein Quantitation Kit, Life Technologies, Denmark) and protein purities (scanning of stained gels) were determined. 

### Synthetic peptides

Synthetic overlapping peptides (15 or 18-mers) covering the complete primary structure of Ag85B, ESAT-6 and TB10.4 were synthesized by standard solid-phase methods on a SyRo peptide synthesizer (MultiSynTech, New England) at the JPT Peptide Technologies (Berlin, Germany) or GenScript (New Jersey, USA). Peptides were lyophilized and stored dry at −20°C until reconstitution in PBS and DMSO (1:1 ratio).

### Histopathological analyses

Lungs were removed post mortem at week 6, 12, and 30 after aerosol infection with *M.tb.* (six mice per group per time-point). The right lung lobe of each mouse was fixed by immersion in 10% neutral-buffered formalin and processed for histological examination. Sections were stained using haematoxylin and eosin and by the Ziehl-Neelsen method and were evaluated without prior knowledge of stage of infection or of treatment group. Lesions were quantified using computer-aided histomorphometry (Palm®robo software, version 1.2.3; Palm Microlaser Technologies AG Ltd, Bernried, Germany). 

### Vaccine evaluations

#### Pre-exposure

Antigens (5 μg) were mixed with CAF01 [28] (except exp. no. 1 and 2 in Table S1 which received the DDA/MPL adjuvant [52]. Mice were vaccinated with 200 μl of the formulation subcutaneously at the base of the tail three times with two weeks interval between each immunization. Negative control mice received three equivalent doses of CAF01. Two weeks after the last immunization, mice were bled and peripheral blood mononuclear cells (PBMCs) were used for immunological analysis as described below. Ten weeks after the first vaccination, virulent *M.tb*. Erdman were delivered via the respiratory route at approximately 100 CFU per mouse (see Figure 2 for experiment outline) with an inhalation exposure system (GlasCol, Indiana, USA). Six mice per group were killed at week 6 post-infection (p.i) (except for exp. no. 3 and 6 in Table S1 which was killed at week 10 and 24 p.i., respectively). The numbers of bacteria in the lung were determined by serial three-fold dilutions of individual organ homogenates in duplicate on 7H11 medium supplemented with PANTA™ (Becton Dickinson, San Diego, USA). Colonies were counted after 2–3 weeks of incubation at 37°C.

#### Post-exposure

Mice were infected via the aerosol route with virulent *M.tb*. Erdman at approximately 50 CFU per mouse. At week 6 p.i. mice were given isoniazid (0.1 g L−1) and rifabutin (0.1 g L−1) (Becton Dickinson) in the drinking water *ad libitum* for six weeks. Immunizations were commenced at week 10 after infection and given three times with three weeks interval unless otherwise stated (see Figure 2 for outline of the experimental setup). Control groups received CAF01 alone or saline and we did not observe any differences in bacterial load when comparing these two control groups (data not shown). All studies were performed using recombinant proteins for vaccine antigens except for the experiment in Figure 5A, where vaccination were done using peptide pools spanning the entire sequence of ESAT-6 or TB10.4. Immune responses were measured one week after the last immunization at week 17 p.i. as described below. Mice were euthanized around week 40 p.i. unless otherwise stated, and numbers of bacteria in the lung were determined by plating serial three-fold dilutions of individual organ homogenates in duplicate on 7H11 medium supplemented with PANTA™ (Becton Dickinson, San Diego, USA). Colonies were counted after 2–3 weeks of incubation at 37°C. The detection limit for the whole organ bacterial counts is 1 CFU based on the plating of undiluted organ homogenates (with the exception of the experiment depicted in Figure 4 and 7B where the detection limit was 10 CFU). In cases where no bacteria were detected, the CFU values have been arbitrarily set at the detection limit. 

#### Therapeutic

Mice were infected via the aerosol route with approximately 50 CFU per mouse. Immunization were commenced at week 6 and given three times with two weeks interval. Mice were vaccinated with either ESAT-6 formulated in CAF01 or CAF01 alone as a control. The numbers of bacteria in the lungs were enumerated at week 21 p.i. by serial three-fold dilutions of individual organ homogenates as described above. 

### Cell preparation, ELISA and flow cytometric analyses

Peripheral blood mononuclear cells (PBMCs) and lung lymphocytes from perfused lungs were isolated, cultured and assessed for immune responses as previously described [29,44,53]. PBMCs were purified on a density gradient of mammal Lympholyte® cell separation media (Cedarlane Laboratories Inc., Canada) according to the manufacturer’s protocol, and followed by two washing procedures using RPMI. Lung lymphocytes were obtained by passage of lungs through a 100 µm nylon cell strainer (BD Pharmingen, USA) followed by two washing procedures using RPMI. Cells were cultured in sterile microtiter wells (96-well plates; Nunc, Denmark) containing 2×10^5^ cells/well for ELISA or 1-2×10^6^cells/well for flow analyses. Both ELISA and flow cultures were in 200 µl of RPMI-1640 supplemented with 5 x 10^-5^ M 2-mercaptoethnaol, 1% pyruvate, 1% HEPES, 1% (v/v) premixed penicillin-streptomycin solution (Invitrogen Life Technologies), 1 mM glutamine, and 10% (v/v) fetal calve serum (FCS) at 37°C/5% CO_2_ and re-stimulated with antigen at 2 µg/ml.IFN-γ levels were measured in supernatants after 72 hours incubation at 37°C by ELISA as previously described [44]. For intracellular staining of cytokines (ICS) cells from blood or lungs of mice were stimulated for 1 hour with 2 µg/ml of antigen and subsequently incubated for 5 h at 37°C with 10 µg/ml brefeldin A (Sigma-Aldrich, Denmark) at 37°C. Cells were washed in FACS buffer (PBS containing 0.1% sodium azide and 1% FCS) before staining with a combination of the following rat anti-mouse antibodies PerCP-Cy5.5-anti-CD8α (53-6.7, RM4-5), APC-Cy7-anti-CD4 (GKI.5), and FITC-anti-CD44 (IM7). Cells were washed with FACS buffer before fixation and permeabilization using the BD Cytofix/Cytoperm™ (BD, San Diego, CA, USA) according to the manufacturer's protocol before staining intracellular with PE-Cy7-Anti-IFN-γ (XMG1.2), PE-anti-TNF-α and APC-anti-IL-2 (JES6-5H4). After washing, cells were suspended in PBS/or formaldehyde solution 4% (w/v) pH 7.0 (Bie & Berntsen, Denmark) and samples were analyzed on a six-color BD FACSCanto flow cytometer (BD Biosciences, USA). Responses were analyzed using Flowjo Software (© Tree Star, Asland, OR, USA) followed by Pestle and Spice software [54] by Boolean gating of IFN-γ, TNF-α and IL-2 (only IFN-γ and IL-2 in Figure 3A) positive CD4 CD44^high^ (gating sequence: singlets, lymphocytes, CD4 vs FSC-A, CD44 vs cytokine). The cytokine responsive cell populations divided according to their cytokine profile was reported either as total % cytokine positive cells (i.e. the sum of all the subpopulations as in Figure 3A and 5C) or with the representation of each individual sub populations (as in Figure 4A). The background (BG) levels of cytokine production measured in media stimulated cells were deducted (BG<0.5%).

### Meta-analysis

The meta-analysis was generated based on fifteen independent experiments with a total number of 726 mice. Each dot in Figure 6 corresponds to a Δlog_10_ protection value for one individual mouse where differences in the overall CFU level in the different experiments were normalized. This was done by subtracting the CFU value of the individual vaccinated by from the median CFU level for the group of control animals in each experiment. Each data point was therefore calculated as follows: median log_10_CFU_controlgroup_ −log_10_CFU_vaccinated animal_. To take the variation of individual control animals into consideration for the statistical analysis, the same subtraction was done for each individual control animal (median log_10_CFU_controlgroup_ −log_10_CFU_control animal_ and the overall data set analyzed by a Kruskal-Wallis statistical analysis. The analysis in total represent 520 data points of vaccine efficacies in the animal post-exposure model of which 40 data points, was calculated based on the original experiments previously reported in the publication by Aagaard et al. [10]. 

### Genome Expression Profiling of MTB using Two-Step Multiplex real time RT-PCR

Frozen lungs from TB-infected mice were homogenized in Trizol containing 20 μg/ml acrylamide. After centrifugation, the debris with unbroken *M.tb.* bacilli was resuspended in Trizol and the *M.tb.* bacilli were disintegrated by bead-beating for 30 seconds at speed 6, 3x (*Fastprep24 bead-beater, MP Biomedicals*) using 0.1mm zirconia/silica beads (*Biospec*). Total RNA was isolated using RNeasy kit (Qiagen) and two off-column RQ1 DNase treatments (Promega), and finally resuspended in 50 μl RNase-free water (Ambion).

#### First strand cDNA synthesis

50ng of each RNA sample was taken into two separate cDNA synthesis reactions (RT+ and RT-) to control for DNA contamination. To each sample, 0.5 µl Exo-resistant Random Primer (Fermentas), 1µl 10 mM dNTPs (Fermentas R0193) and 3.5 µl of Nuclease Free Water (Ambion AM9938) were added for a total of 10 µl. This mix was incubated for 3 minutes at 70 °C and then placed on ice. Reverse Transcription (RT)+ cocktail containing 4 µl 5X Maxima RT Buffer (Fermentas), 0.5 µl Ribolock RNase-Inhibitor (Fermentas), 0.5 µl Maxima RT enzyme (Fermentas), and 3.0 µl Nuclease Free Water for a total of 10 µl were prepared together with the RT- cocktail where RT enzyme was substituted with water. These cocktails were scaled up for multiple samples and 10 µl aliquots were added to each RT+ or RT- sample prepared above. Reverse transcription was carried out for 1 hour at 50 °C, heat inactivated at 95 °C for 2 minutes, and then held at 4 °C. 

#### Multiplex Pre-amplification PCR

PCR was performed as described previously [10,55]. Briefly, equal volumes of outflanking forward and reverse primers were mixed together. The final concentration of the primers in the amplification reaction was 52 nM. Each pre-amplification PCR reaction consisted of 3.0 µl 10X Advantage 2 Buffer (Clontech), 0.6 µl 10 mM dNTPs (Fermentas), 23.8 µl Primer Mix, and 0.6 µl Advantage 2 Polymerase (Clontech). 2 µl of RT+ or RT- cDNA was then added for a total of 30 µl. Controls of water and two aliquots of 10^4^ gene copy number of H37Rv genomic DNA were also amplified. Samples were denatured at 95 °C for 5 minutes. Fifteen cycles of PCR were run at 95 °C for 30 seconds, 60 °C for 20 seconds, and 68 °C for 1 minute. Complete database with all available validated TaqMan sets can be found at ftp://smd-ftp.stanford.edu/tbdb/rtpcr/taqman_oligos.fa. Sequences and design of PCR primer/probe sets can be found at http://genes.stanford.edu/technology.php and http://www.tbdb.org/rtpcrData.shtml. Each 10 µl qRT-PCR reaction contained 300 nM of forward and reverse primers and 100 nM of the probe and was run on the 384-well format platform using Roche Lightcycler 480. Taq DNA Polymerase was activated at 95 °C for 5 minutes. Then 40 cycles of PCR followed: 95 °C for 30 seconds and 60 °C for 30 seconds. The data were analyzed using Roche’s Second Derivative High Confidence algorithm on the Roche LightCycler Software.

#### Normalization and Data Analysis

For normalization, we adapted a quantile normalization developed for DNA microarray expression analysis, where data-driven methods have become the standard for most experimental designs [56]. Quantile normalization is based on the assumption that on average, the distribution of gene transcript levels within the cell remains nearly constant across samples. A quantile is a measure that allows assessing the degree of spread in a data set. Quantile normalization adjusts the overall expression levels so that the distribution for all samples is equal. First, median cycle threshold (Ct) value was calculated for all genes for each sample and then median of the median individual Ct values for all samples calculated. Then, quantile normalization was applied between samples, assuring that each sample has the same distribution of expression values as all of the other samples to be compared. A similar approach has been previously described for microarray normalization [57]. 

### Statistical analysis

For the pre-exposure data which follows a normal distribution protective efficacies of the vaccines (log_10_ CFU´s) were compared by one-way analysis of variance (ANOVA) followed by Tukey´s multiple comparison test of the means. For the post-exposure evaluation of vaccines, we generally found larger variation within the groups and the data points did not follow a normal distribution, hence non-parametric test were used for these sets of experiments. Protective efficacies (log_10_ CFU´s) were compared by a non-parametric Mann-Whitney U-test when comparisons were done between two groups e.g. between ESAT-6 vaccinated and control group in Figure 7B and a Kruskall-Wallis for comparison of medians between several groups e.g. in Figure 5 and 6. For all tests, p-values <0.05 were considered statistically significant.

## Supporting Information

Table S1 **Bacterial levels following pre-exposure vaccination with ESAT-6 and TB10.4 containing vaccines.** ^a^Bacterial load was determined by culturing lung homogenate and enumerating the bacteria. Bacterial levels are expressed as mean log_10_ CFU± standard error of the mean. ^b^ Groups of mice were vaccinated three times with H4, H28, H1 or H56 formulated in CAF01. In Exp. and 2, the DDA/MPL adjuvant was used instead of CAF01. Control group were vaccinated three times with CAF01 alone. ^c^ not determined in the experiment. ^d^**p<0.01, ^e^***p<0.001 vaccine group compared to control group, one-way ANOVA, Tukey’s multiple comparison test.
(DOCX)
