# Supplementary material for: ESAT-6 (EsxA) and TB10.4 (EsxH) Based Vaccines for Pre- and Post-Exposure Tuberculosis Vaccination
Source: PLoS One. 2013 Dec 12;8(12):e80579. doi: 10.1371/journal.pone.0080579 (PMC3861245; doi:10.1371/journal.pone.0080579)
Supplement: Table S1 — Bacterial levels following pre-exposure vaccination with ESAT-6 and TB10.4 containing vaccines. aBacterial load was determined by culturing lung homogenate and enumerating the bacteria. Bacterial levels are expressed as mean log10 CFU± standard error of the mean. b Groups of mice were vaccinated three times with H4, H28, H1 or H56 formulated in CAF01. In Exp. and 2, the DDA/MPL adjuvant was used instead of CAF01. Control group were vaccinated three times with CAF01 alone. c not determined in the experiment. d**p<0.01, e***p<0.001 vaccine group compared to control group, one-way ANOVA, Tukey’s multiple comparison test. (DOCX) [file pone.0080579.s001.docx]

Table S1. Bacterial levels^a^ following pre-exposure vaccination with ESAT-6 and TB10.4 containing vaccines

|  | Vaccine group^b^ | | | | |
| --- | --- | --- | --- | --- | --- |
| Experiment no. | control | H4 | H28 | H1 | H56 |
| 1 | 5.59 ± 0.09 | 4.59 ± 0.08***^e^ | n.d.^c^ | 4.47 ± 0.05*** | n.d. |
| 2 | 5.64 ± 0.04 | 5.16 ± 0.08*** | n.d. | 5.03 ± 0.10*** | n.d. |
| 3 | 4.80 ± 0.14 | n.d. | n.d. | 3.92 ± 0.13**^d^ | 3.93 ± 0.15** |
| 4 | 5.26 ± 0.14 | 4.54 ± 0.03*** | 4.46 ± 0.14*** | n.d. | n.d. |
| 5 | 5.47 ± 0.06 | 4.79 ± 0.18** | 4.19 ± 0.07*** | n.d. | n.d. |
| 6 | 5.87 ± 0.08 | n.d. | n.d. | 4.15 ± 0.14*** | 4.79 ± 0.10*** |
| 7 | 6.54 ± 0.11 | n.d. | n.d. | 5.17 ± 0.16*** | 5.05 ± 0.13*** |
| 8 | 5.05 ± 0.10 | n.d. | n.d. | 4.08 ± 0.22** | 3.83 ± 0.16*** |
| 9 | 4.89 ± 0.07 | n.d. | 3.91 ± 0.16*** | n.d. | 3.81 ± 0.17*** |

^a^Bacterial load was determined by culturing lung homogenate and enumerating the bacteria. Bacterial levels are expressed as mean log_10_ CFU± standard error of the mean. ^b^ Groups of mice were vaccinated three times with H4, H28, H1 or H56 formulated in CAF01. In Exp. and 2, the DDA/MPL adjuvant was used instead of CAF01. Control group were vaccinated three times with CAF01 alone. ^c^ not determined in the experiment. ^d^**p<0.01, ^e^***p<0.001 vaccine group compared to control group, one-way ANOVA, Tukey’s multiple comparison test.
